# Assimilatory N_2_O reduction by *Nostoc* sp. strain MS1 isolated from a river: insights from genome and ^15^N tracer analysis

**DOI:** 10.3389/fmicb.2026.1759539

**Published:** 2026-03-16

**Authors:** Kazumi Suda, Toshikazu Suenaga, Soichiro Matsuzaki, Shohei Riya, Kento Ishii, Manami Nomachi, Hirotsugu Fujitani, Satoshi Tsuneda, Kartik Chandran, Akihiko Terada

**Affiliations:** 1 Department of Applied Physics and Chemical Engineering, Tokyo University of Agriculture and Technology, Koganei, Tokyo, Japan; 2 Department of Chemical Engineering, Hiroshima University, Higashihiroshima, Hiroshima, Japan; 3 Department of Life Science and Medical Bioscience, Waseda University, Tokyo, Japan; 4 Department of Earth and Environmental Engineering, Columbia University, New York, NY, United States; 5 Institute of Global Innovation Research, Tokyo University of Agriculture and Technology, Fuchu, Tokyo, Japan; 6 Research Division of Nutrient Management, Advanced Research Center for One Welfare, Tokyo University of Agriculture and Technology, Fuchu, Tokyo, Japan

**Keywords:** ^15^N tracer, cyanobacteria, fluorescence-activated cell sorter, nitrous oxide, *Nostoc* sp.

## Abstract

Direct evidence for the assimilation of nitrous oxide (N₂O), a potent greenhouse gas, by freshwater cyanobacteria has been lacking. Here, we report a cyanobacterium, isolated from a nitrogen-polluted river, that fixes N_2_O via dinitrogen (N_2_) gas by nitrogenase activity. N_2_O-reducing bacteria were enriched from river samples, under alternating light/dark conditions in the presence of atmospheric N_2_ and the absence of oxygen (O_2_), followed by isolation using fluorescence-activated cell sorting. The isolated strain, *Nostoc* sp. strain MS1 (NIES-4466), consists of moniliform coccoid cells and is phylogenetically affiliated with the genus *Nostoc*. A high-quality draft genome of strain MS1 revealed the presence of nitrogenase genes encoding the MoFe protein but the absence of N_2_O reductase genes, i.e., clades I, II, and III *nosZ*. When incubated in a He (95%)/CO_2_ (5%) atmosphere with 0.01% ^15^N-labeled N_2_O, the cells exhibited elevated ^15^N content relative to natural abundance (0.36%). The degree of ^15^N incorporation positively correlated with ethylene production from acetylene, implicating nitrogenase in N_2_O assimilation by strain MS1. While replacing He with N_2_ reduced N_2_O uptake, likely due to substrate competition, N_2_O consumption activity persisted, suggesting that freshwater cyanobacteria can function as an N_2_O sink. These findings, supported by genomic and ^15^N tracer analyses, highlight the previously unrecognized role of cyanobacteria in mitigating N₂O emissions in freshwater environments.

## Introduction

Nitrous oxide (N_2_O) is a highly potent greenhouse gas, 273 times more powerful than carbon dioxide (Shukla et al., 2022) and a long-lived ozone-depleting substance (Ravishankara et al., 2009). The primary anthropogenic sources are agricultural croplands, biomass incineration, and wastewater treatment, and the amount released has increased by 30% over the past four decades (Tian et al., 2020). Given the increase in global population in the 21st century, atmospheric N_2_O concentrations are expected to continue rising (Davidson and Kanter, 2014). Many strategies to reduce N_2_O emissions have been investigated, including the application of biofilms or N_2_O-reducing bacteria, and have been successful in reducing N_2_O emissions from environments such as engineered ecosystems and croplands (Itakura et al., 2013; Yoon et al., 2017; Maeda et al., 2025).

Although there are multiple N_2_O production pathways relevant to nitrogen cycling in natural and engineered environments (Stein, 2019; Prosser et al., 2020), N_2_O consumption as an N_2_O sink is mainly mediated by denitrifying and non-denitrifying bacteria harboring a functional gene (*nosZ*) for N_2_O reductase (Nos) (Zumft, 1997; Zumft and Kroneck, 2007; Pauleta et al., 2013). Pioneering studies have implicated phylogenetically diverse bacteria with *nosZ* as N_2_O sinks (Sanford et al., 2012; Jones et al., 2013; He et al., 2025). Leveraging these N_2_O-reducing bacteria for N_2_O consumption shows promise in N_2_O hotspots. The downside of this strategy lies in the Nos susceptibility to various environmental conditions, especially in the presence of oxygen (O_2_) and other nitrogen oxides such as nitrate and nitrite. Therefore, the activities of N_2_O-reducing bacteria as N_2_O sinks are undermined in aerobic-microaerobic environments. Further investigation of the physiology of N_2_O-consuming microbes, which includes reduction and assimilation, is required (Yoon et al., 2025).

Cyanobacteria are broadly distributed in natural environments (Nawaz et al., 2025) and contribute to primary production by fixing inert nitrogen in marine and freshwater environments (Bauersachs et al., 2010; Howarth et al., 1988; Zehr, 2011). Some nitrogen-fixing cyanobacteria, e.g., *Trichodesmium* spp. (Farias et al., 2013; Li et al., 2024), and Pseudomonadota (formerly Proteobacteria), e.g., *Azotobacter vinelandii*, can assimilate nitrogen from N_2_O as a nitrogenase substrate (Mozen and Burris, 1954; Farias et al., 2013). Natural marine (Saxena et al., 2025) and freshwater environments (Si et al., 2023) harbor microbial communities that exhibit N_2_O-uptake activities. These discoveries support the identification of a new N_2_O sink, offering a new paradigm for more effective use of N_2_O-assimilating microbes to mitigate N_2_O emissions. Despite the broad ecological niches of these microorganisms that assimilate N_2_O, to our knowledge, no studies have reported an isolated freshwater N_2_O-assimilating cyanobacterium. Given that the amount of N_2_O emissions from freshwater environments has increased with anthropogenic activities (Kroeze et al., 2010), and N_2_O emissions from freshwater environments are two orders of magnitude higher than those from sea environments (Shukla et al., 2022), cyanobacteria capable of assimilating N_2_O are likely to exist in freshwater environments.

Despite the potential for freshwater N_2_O-assimilating bacteria to act as N_2_O sinks, the mechanisms by which N_2_O is assimilated and the ecological niches occupied by these bacteria remain unresolved. *In vitro* research on nitrogenases in rhizobacteria affiliated with the phylum Pseudomonadota and *Klebsiella pneumonia* indicates the conversion of N_2_O to dinitrogen (N_2_) (Hoch et al., 1960; Jensen and Burris, 1986). ^15^N tracer analysis demonstrated that *Pseudomonas stutzeri* converts N_2_O into N_2_ via respiratory reduction, followed by N_2_ fixation and NH_4_^+^ incorporation into microbial biomass as assimilation (Desloover et al., 2014). One inconsistent result showed that *A. vinelandii* directly assimilates N_2_O into cellular biomass without producing N_2_ by nitrogenase (Yamazaki et al., 1987). These conflicting results indicate that N_2_O consumption mechanisms are multiple, with assimilation occurring both via N_2_ and via bypassing N_2_.

To leverage N_2_O-assimilating bacteria as an N_2_O sink, it is vital to understand how O_2_ and N_2_ partial pressures affect N_2_O uptake. Whereas N_2_O reductase (Nos) in canonical denitrifying bacteria is inactivated under aerobic conditions, some cyanobacteria may assimilate N_2_O in the presence of O_2_, likely because they harbor heterocysts (Ermakova et al., 2013). N_2_O and N_2_ compete for nitrogenase (Repaske and Wilson, 1952; Rivera-Ortiz and Burris, 1975), and the preferred nitrogen compound, i.e., N_2_O vs. N_2_, for nitrogenase has not yet been identified.

This study, therefore, hypothesized that: (1) a freshwater environment harbors cyanobacteria capable of taking up N_2_O; (2) N_2_O is converted into N_2_ to be fixed for assimilation (or is directly transformed to ammonia for assimilation); and (3) a cyanobacterium exists with higher O_2_ tolerance than the canonical denitrifying N_2_O-reducing bacterium. To test these hypotheses, we enriched a river sample devoid of O_2_ and external organic carbon, and isolated a bacterium from the enriched biomass. A high-quality draft genome of the isolate was used to holistically understand its metabolic potential, especially focusing on N_2_O uptake in the absence of O_2_ and nitrogen, and to propose a mechanism for nitrogenase’s tolerance to O_2_. Finally, a ^15^N tracer study was performed to systematically investigate the effects of O_2_ and N_2_ on N_2_O uptake by the isolated cyanobacterium.

## Materials and methods

### Biomass collection and enrichment

Water from the Hokota River flowing into Lake Kitaura in Ibaraki Prefecture, Japan (36.143906, 140.512936) was collected as an inoculum to isolate N_2_O-assimilating cyanobacteria. The sampling site is located near agricultural cropland that receives high nitrogen loading from fertilizer application, and the river is highly contaminated with ammonia and nitrate (Yoshinaga et al., 2011). River water (30 mL) was aliquoted into a 100 mL vial. After N_2_ purging to remove O_2_ from the water, each vial was sealed with a butyl rubber stopper. Carbon dioxide (CO_2_) was added to the atmospheric gas, resulting in a gas composition of N_2_ (75%), O_2_ (20%), and CO_2_ (5%). No other nutrients were provided. During the incubation (40 days) to enrich cyanobacteria, the vials were intermittently exposed to fluorescent light at 12 h intervals. After 40 days of incubation under light exposure with CO_2_, 0.1 mL of biomass suspension was transferred to 30 mL of BG_0_11 medium. This was then subsequently incubated under the following headspace conditions: He (94.7%), CO_2_ (5%), and N_2_O (0.3%) using a 100 mL vial. The N_2_O concentration was monitored with GC-ECD for 68 days, and the biomass was used for isolation.

### Isolation and incubation of cyanobacteria

**Figure 1:**
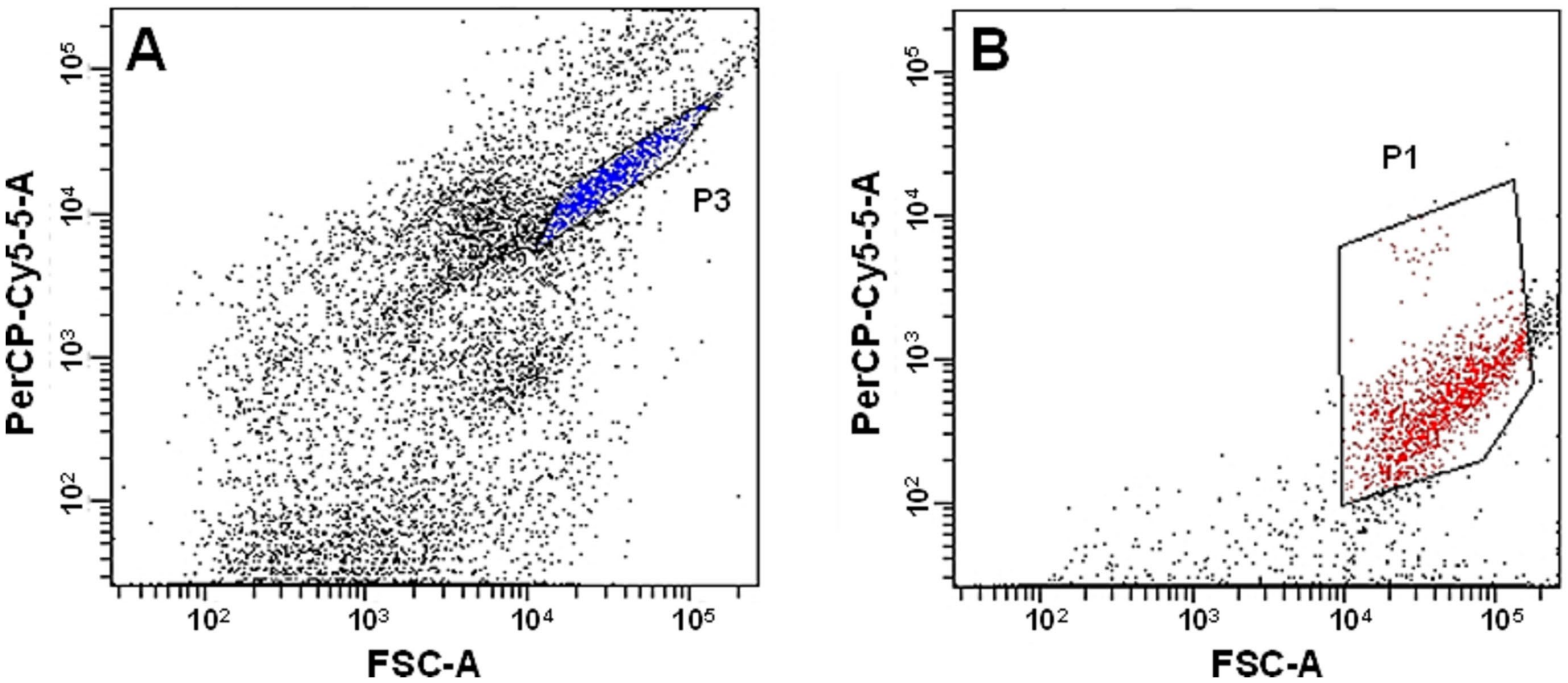
Separation of cyanobacteria from the enriched biomass by a fluorescence-activated cell sorter (FACS) using relative fluorescence of chlorophyll as a function of relative cell size. **(A)** First-round application and **(B)** the second-round application. The blue plots in area P3 in panel **A** were sorted, followed by microscopic inspection. The red plots in area P1 in panel **B** were sorted based on the observation of moniliform cyanobacteria cells under light microscopy.

Isolation of the cyanobacteria enriched in the vial was conducted by a fluorescence-activated cell sorter (FACS Aria II, BD, Franklin Lakes, NJ, United States). As in a previous study, the biomass was ultrasonicated (Sonifier II model 150, Branson, Danbury, CT, United States) (Fujitani et al., 2014), and filtered through a 35 μm filter to remove large biomass aggregates. The filtrate flow rate in the cell sorter was adjusted to 200–300 events/s in single-cell mode, yielding an estimated 10^5^–10^6^ sorted cells per sample. Because cyanobacteria often form moniliform filaments (i.e., a bead-shaped morphology) and contain chlorophyll, forward scatter (FSC) and peridinin-chlorophyll-protein complexes (PerCP-Cy5.5) were used to separate cells by size and autofluorescence intensity, respectively. The PerCP-Cy5.5 was excited by a laser at an excitation wavelength of 488 nm, and fluorescence was observed at an emission wavelength of 676 nm. The dot-plot area between FSC and PerCP-Cy5.5 was designated onto a two-parameter histogram. The cyanobacterial cells in the dot plot areas, where relatively homogeneous cell morphologies were observed (P3 and P1 in Figure 1), were individually allocated to two 96-well plates for FACS analysis.

Cells sorted onto the 96-well plate were incubated with a 12 h light–dark cycle under aerobic and anoxic conditions to investigate whether redox conditions affect cell growth. Each plate was filled with a headspace gas comprising either N_2_ (75%)/O_2_ (20%)/CO_2_ (5%) or N_2_ (95%)/CO_2_ (5%). The cells grew successfully irrespective of the headspace gas compositions. If the optical density in a well increased because of cell growth, the contents were transferred to a test tube for morphological observation and 16S rRNA gene sequencing.

### Phylogenetic analysis of the isolated cyanobacterium

After incubation, the cell suspension was collected from the test tube by centrifugation at 5,000 rpm for 2 min. DNA was extracted from the centrifuged cell pellet with the Fast DNA Spin kit (MP Biomedicals, Irvine, CA, United States). The extracted DNA concentration was measured and adjusted to 2 ng/μg with a spectrophotometer (Nanodrop 2000C, Thermo Fisher Scientific, Waltham, MA, United States), followed by the amplification of the 16S rRNA gene with the universal primer set 341f-907r (Muyzer et al., 1995). The PCR conditions, cloning procedures, and methods for 16S rRNA gene identification are described in the Supplementary material. To confirm if an isolated cyanobacterium possessed a functional gene encoding N_2_O reductase (*nosZ* gene), PCR was used to detect the *nosZ* gene clade I (Henry et al., 2006) and clade II (Jones et al., 2013) types, as previously described (Kinh et al., 2017). Finally, 16S rRNA gene sequencing identified the isolated strain as the cyanobacterium, *Nostoc* sp. strain MS1, and it has been deposited in the NIES collection as NIES-4466.

### Construction of *Nostoc* sp. strain MS1 genome

The genomic DNA of *Nostoc* sp. strain MS1, was extracted with a phenol-chloroform extraction technique (Butler, 2012) followed by purification with cetyltrimethylammonium bromide. RNA in the genomic DNA was decomposed with RNaseA (TaKaRa Bio, Inc., Shiga, Japan). The construction of the genome library was described previously (Yasuda et al., 2020). In short, the genome library was prepared with a 1D ligation sequencing kit (SQK-LSK-109; Oxford Nanopore Technologies Ltd., Oxford, United Kingdom) and sequenced on the MinION Mk1B with an R9.4 flow cell (FLO-MIN106; Oxford Nanopore Technologies Ltd., Oxford, United Kingdom). The attained sequence quality was confirmed by NanoPlot (ver. 1.20.0) (De Coster et al., 2018), where the adaptor sequences, low-quality reads (<Q10), header (75 bp), and short reads (<100 bp) were removed using Porechop (ver. 0.2.4).^1^ According to the manufacturer’s protocol, short-read sequencing was performed with the NEBNext Ultra DNA Library Prep Kit for Illumina (NEB, MA), and index codes were added to attribute sequences to each sample. Subsequently, 150 bp paired-end sequencing with NovaSeq 6000 (Illumina, CA) was conducted by a sequencing service (Novogene, Beijing, China). The adapter sequences and low-quality reads (<Q20) were removed using Trim Galore (ver. 0.6.5; http://www.bioinformatics.babraham.ac.uk/projects/trim_galore/). Each genome sequencing by MinION and NovaSeq had 426× and 97× depth of coverage, respectively. The consensus sequence was assembled with Unicycler (ver. 0.4.7) (Wick et al., 2017) as a hybrid of the long- and short-reads. The genome completeness (95%) was assessed with BUSCO v1 (Simão et al., 2015). The missing marker genes [*serS* (COG0172) and *rpsO* (COG0184)] were manually detected in the annotated sequence, ensuring 100% completeness. The coding region of a gene was detected and annotated using DFAST (ver. 1.1.5) (Tanizawa et al., 2016) and KofamKOALA (ver. 2020-01-06) (Aramaki et al., 2019), respectively. As some *Nostoc* spp. excrete toxins [e.g., microcystins (Oksanen et al., 2004)], the presence of genes encoding toxins were confirmed by Pathogen Finder (ver.1.1) (Cosentino et al., 2013). Default parameters were used for all software unless otherwise specified.

### N_2_O fixation ability measured by ^15^N-labeled N_2_O (N_2_O fixation test)

We selected the isolated freshwater cyanobacterium, i.e., *Nostoc* sp. strain MS1, and other cyanobacteria deposited in the NIES collection (https://mcc.nies.go.jp/index_en.html, Table 1) to confirm the potential for N_2_O uptake by freshwater cyanobacteria. A ^15^N tracer method was used to investigate N_2_O uptake potentials of the isolated and nitrogen-fixing cyanobacteria, and to elucidate a mechanism for N_2_O uptake. The tested cyanobacteria were incubated in a vial spiked with ^15^N-labeled N_2_O (^46^N_2_O). The amount of N_2_O assimilated and converted by the tested cyanobacteria was quantified by the ^15^N content in the cells and the accumulated headspace N_2_. The cultivated media (30 mL) listed in Table 1 were added to a 100 mL vial, followed by purging with He gas for 3 min, vacuuming the headspace, and sealing the vial. The mixed gas, consisting of 95 mL He, 5 mL CO_2_, and 0.1 mL 10% ^46^N_2_O, was introduced, and the vial was autoclaved. The strain of each cyanobacterium was pre-incubated with the recommended cultivation medium described in Table 1, and a cell suspension was adjusted to an OD_730_ of 0.1, and 0.2 mL of the cell suspension was added to the vial. The cyanobacteria were incubated for 12 days at 25 °C, 80 rpm, and a light intensity of 5.5 W/m^2^, with a 12 h light: dark cycle. In parallel, a blank vial devoid of cell suspension was prepared to attain the background value for ^15^N assimilation by the cyanobacterial cells. Each experimental run was performed in triplicate. The total ^15^N content and percentage were quantified by isotope-ratio mass spectrometry (IR-MS; Flash Flash2000-DELTAplus Advantage conFloI System; Thermo Fischer, Waltham, MA). The preparation procedures are further described in the Supplementary material.

**Table 1:** Cyanobacteria used in the N_2_O incorporation test in this study.

NIES No.	Strain name	Cultivation medium	Accession No. of genome or 16S rRNA gene
4466	*Nostoc* sp. strain MS1 (isolated in this study)	BG11_0_	AP023441–AP023446
21	*Anabaenopsis circularis* (G.S. West) Woloszynska & Miller	BG11_0_	AP018174
22	*Calothrix brevissima* G.S. West	BG11_0_	AP018207
50	*Aulosira laxa* Kirchner ex Bornet & Flauhault	BG11_0_	AP018307
2094	*Nostoc* sp.	BG11_0_	16S rRNA (LC190509)
2111	*Nostoc* sp.	BG11_0_	AP018184
2116	*Oscillatoria neglecta* Lemmermann	BG11_0_	16S rRNA (AB003168)
2118	*Leptolyngbya* sp.	CB	16S rRNA (AB003163)
2119	*Phormidium ambiguum* Gomont	BG11_0_	MRCE00000000
2130	*Scytonema* sp.	BG11_0_	MRCF01000179
3276	*Plectonema boryanum* Gomont	Modified BG11_0_	—
3754	*Fischerella* sp.	BG11_0_	AP017305

### Confirmation of nitrogen fixation by an acetylene reduction assay

In parallel with the ^15^N tracer method, an acetylene reduction assay (Hardy et al., 1973) was performed to investigate the correlation between the nitrogen fixation rate and the degree of ^46^N_2_O uptake. The experimental setup was almost the same as for the ^15^N tracer experiment, but differed in the initial headspace gas composition (85 mL He, 5 mL CO_2_, and 10 mL acetylene). The amount of ethylene produced over time in each vial was measured by GC-FID (see Supplementary Table S1 for conditions). The molar conversion ratio of ethylene production with nitrogen (and/or N_2_O) fixation was applied C_2_H_4_:N_2_ = 3:1, which was used a previously proposed stoichiometry (Hardy et al., 1973). Although the molar conversion may not reflect a real situation, and other molar ratios may be expected (Nohrstedt, 1983), this method is still valid and has been widely employed in previous nitrogen fixation research (Hardy et al., 1973; Chalk et al., 2017). On day 12 of the incubation, the biomass was filtered through a glass fiber membrane filter (GF/F, Whatman, GE Healthcare, Chicago, IL, United States) and used to calculate a specific nitrogen fixation rate.

### O_2_ effect on N_2_O uptake by *Nostoc* sp. strain MS1

The effect of O_2_ on the amount of N_2_O uptake by the isolated bacterium *Nostoc* sp. strain MS1 was investigated. Four runs at different O_2_ partial pressures (P_O2_) were prepared. A 100 mL vial received 30 mL BG11_0_ medium (Rippka et al., 1979), was purged with He for 3 min, vacuumed, and sealed. Each vial headspace was replaced with different mixtures of He: O_2_: CO_2_ [95:0:5 (mL) in Run 1; 90:5:5 in Run 2; 75:20:5 in Run 3; and 65:30:5 in Run 4]. Then, 0.1 mL of 10% ^46^N_2_O gas was added to each vial to adjust the N_2_O concentration to 100 ppm, and the vials were autoclaved. Each vial then received 0.2 mL of a preincubated cell suspension of *Nostoc* sp. strain MS1 with an OD_730_ of 0.1. The incubation condition and sampling procedure for the batch test were the same as for the N_2_O uptake test. Each run was conducted in triplicate. At the end of the experiment, the cell suspension was harvested to quantify the ^15^N content in the cells (details in Supplementary material). The amount of ATP in the cell suspension was photochemically quantified using a BacTiter-Glo^™^ Microbial Cell Viability Assay (Promega, Madison, WI) according to the manufacturer’s protocol. Because we observed rapid photobleaching stemming from ATP, a calibration was performed after every five measurements.

### Competitive effect of nitrogen on N_2_O uptake by *Nostoc* sp. strain MS 1

The competitive effect of N_2_ on N_2_O uptake by *Nostoc* sp. strain MS1 was investigated. Three runs were prepared with different headspace gas compositions:

Run A: N_2_ (95 mL) and CO_2_ (5 mL) with 100 ppm of N_2_O gas.
Run B: He (95 mL) and CO_2_ (5 mL) with 100 ppm of N_2_O gas.
Run C: He (95 mL) and CO_2_ (5 mL) with 100 ppm of N_2_O gas, no biomass.

BG11_0_ medium (Castenholz, 1988) with a slight modification (Supplementary Table S2) was prepared to test the nitrogen balance during N_2_O fixation by *Nostoc* sp. strain MS1. The medium was prepared without ammonia, and the *N*-Tris (hydroxymethyl) methyl-2-aminoethanesulfonic acid concentration was reduced by 90%. Modified BG11_0_ medium (30 mL) was added to a 100 mL vial, purged with He for 3 min, and sealed. All vials received 0.1 mL 10% ^46^N_2_O to adjust the gaseous N_2_O concentration to 100 ppm and were then autoclaved. The suspension of *Nostoc* sp. strain MS1 was pre-incubated in an Erlenmeyer flask, and then added to a screw-top tube and centrifuged at 5,000 rpm for 5 min. The pellet was washed twice with 0.02× PBS. The washed cell suspension (1 mL) at an OD_730_ of 0.47 was added to each run except Run C (Control). All the vials were incubated at 80 rpm and 25 °C with light and dark intervals of 12 h each. The headspace N_2_O, O_2_, and ^30^N_2_ concentrations were measured every few days. Dissolved N_2_O concentration was calculated based on the gas–liquid equilibrium (Holtan-Hartwig et al., 2000).

To ascertain if genes encoding N_2_O reductase could have contaminated the liquid phase, the liquid from each vial was retrieved, and DNA was extracted with the Fast DNA Spin kit (MP Biomedicals, Irvine, CA, United States). Extracted DNA was assessed by endpoint PCR to detect *nosZ* clade I and *nosZ* clade II genes, as previously reported (Kinh et al., 2017). The suspension in each vial was filtered with a glass fiber membrane filter (GF/F, Whatman, GE Healthcare) to measure dissolved total nitrogen (DTN), ammonium (NH_4_^+^), nitrite (NO_2_^−^), and nitrate (NO_3_^−^).

### Chemical analysis

DTN was measured using a total organic carbon (TOC) analyzer with a total nitrogen measurement unit (TOC 5000A, Shimadzu, Kyoto, Japan), and NH_4_^+^, NO_2_^−^, and NO_3_^−^ were measured by ion chromatography (ICS-1000, Thermo Fisher Scientific, Sunnyvale, CA, USA) with columns for cations (IonPac CS12A, Thermo Fisher Scientific) and anions (IonPac AS12A, Thermo Fisher Scientific). The amount of total nitrogen and the proportion of ^15^N (^15^N atom%) were measured by IR-MS (see Supplementary material). Gaseous concentrations of N_2_O and O_2_ in the headspace were measured by gas chromatography with an electron capture detector (GC-ECD, GC-14B, Shimadzu, Kyoto, Japan). The conditions for the GC-ECD analysis were described previously (Terada et al., 2013) and are summarized in Supplementary Table S3. Gaseous ^30^N_2_ was measured by quadrupole GC–MS (GCMS-QP2010 Plus, Shimadzu, Kyoto, Japan); conditions are described in Supplementary Table S4. As an indicator of the degree of N_2_O uptake, the ethylene concentration was measured by GC-FID (GC-2014, Shimadzu, Kyoto, Japan) with the conditions described in Supplementary Table S1.

### Statistical analysis

Statistical significance was assessed using one-way analysis of variance (ANOVA) followed by Tukey’s test for multiple comparisons in SPSS Statistics (version 27; IBM Corp., Armonk, NY) and R (version 4.5.2; R Core Team). A significance level in this study was set at *p* < 0.05.

## Results

### Isolation of cyanobacteria

An inoculum of N_2_O-fixing cyanobacteria was collected from the Hokota River in Ibaraki Prefecture, Japan, and enriched with N_2_ (75%), O_2_ (20%), and CO_2_ (5%) for 40 days and subsequently with He (94.7%), CO_2_ (5%), and N_2_O (0.3%) for 68 days. Microscopic observation revealed the filamentous morphology of the cyanobacterium-like microorganisms. Although heterotrophic N_2_O reduction and fixation could not be distinguished, the N_2_O consumption activity was detected (Supplementary Figure S1), suggesting the enrichment of the biomass fixing N_2_O. Cyanobacterial cells were collected from the enriched biomass with a fluorescence-activated cell sorter (FACS). Because the cells in a dot plot area P3 (Figure 1A) did not consist of a single cyanobacterial species (details in Supplementary material), the cell suspensions were subjected to a second round of cell sorting by the FACS system to improve the purity (P1 area in Figure 1B). The second-round FACS and the subsequent aerobic incubation allowed the isolation of individual cyanobacterial strains. We confirmed the predominance of moniliform coccoid cells with identical morphology by light and epifluorescence microscopy (Supplementary Figure S2). No growth of contaminated heterotrophic bacteria was observed, as evidenced by microscopic analysis and by the distinct single peaks in 16S rRNA gene-based Sanger sequencing. In addition, no contamination was detected during the quality check for the registration in the NIES culture collection.

### Phylogenetic analysis of the isolated cyanobacteria

**Figure 2:**
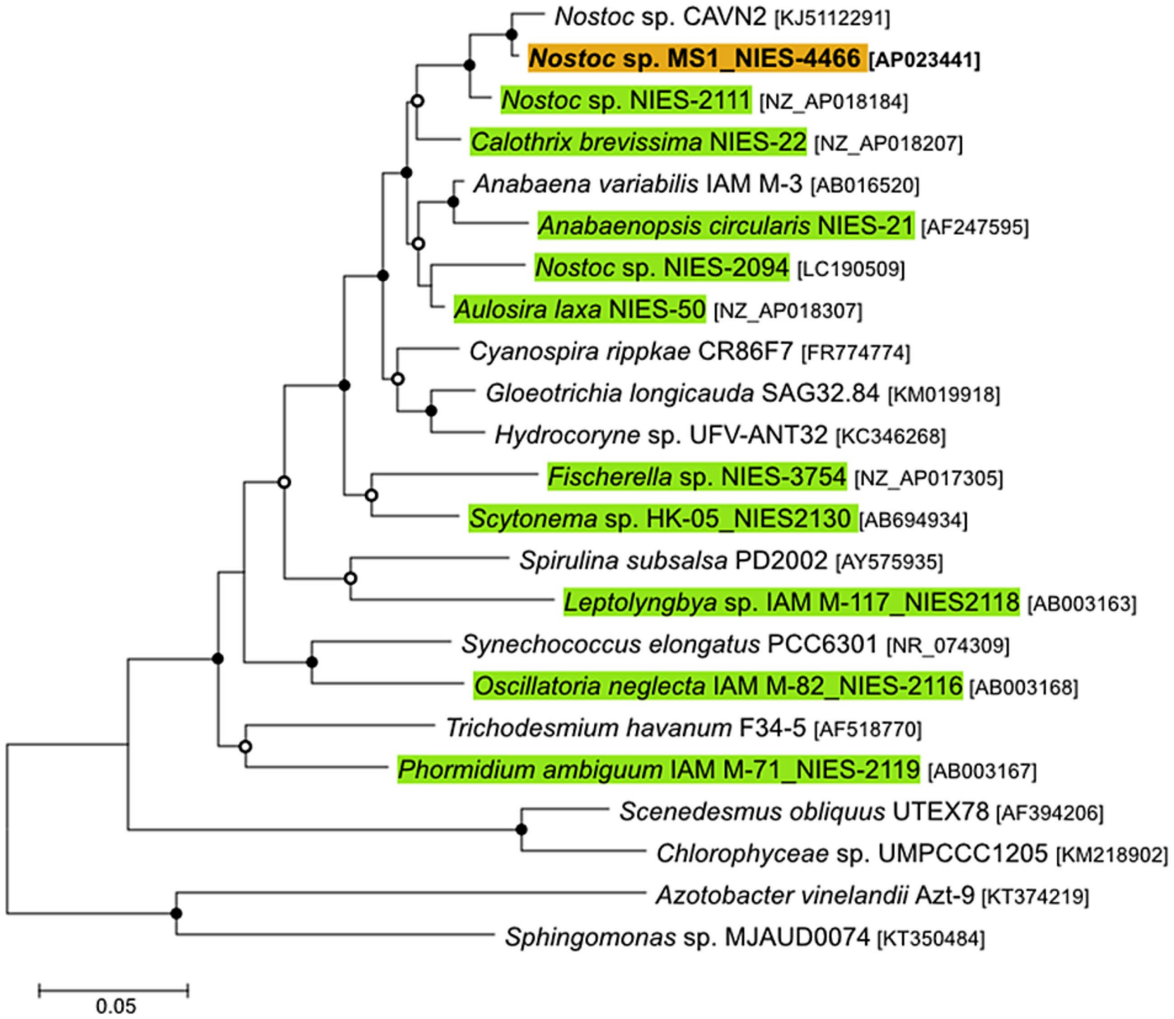
Maximum likelihood phylogenetic tree based on 16S rRNA gene sequences. Open and closed circles at nodes represent bootstrap values from 50 to 75% and 75 to 100%, respectively. The scale bar denotes 5% sequence divergence.

The phylogenetic analysis revealed that the 16S rRNA gene sequence of the isolated bacterium *Nostoc* sp. strain MS1 is identical to *Nostoc* sp. BLAST search revealed the strain proximal to *Nostoc* sp. CAVN2 (percent identity: 98.23%) and *Nostoc* sp. NIES2111 (percent identity: 98.39%) in the *Nostocaceae* family (Figure 2). Furthermore, endpoint PCR with primers for the clade I and clade II *nosZ* genes did not amplify either gene, indicating that the isolated strain does not perform respiratory N_2_O reduction.

### Genome reconstruction of *Nostoc* sp. strain MS1

The quality check (QC)-passed long-read sequencing had a total base number of 3.37 Gbp and a mean length of 19.1 kbp. The QC-passed short-read sequencing totaled 0.770 Gbp. The genome length was 7,901,148 bp, consisting of six circular contigs of 7,141,037 bp; 307,420 bp; 303,628 bp; 58,668 bp; 45,733 bp; and 44,662 bp. The sequence comprised 6,809 coding sequences, a GC content of 40.5%, 8 rRNAs, and 93 tRNAs (determined using DFAST). Functional annotation revealed that *Nostoc* sp. strain MS1 possesses all genes for glycolysis and the Calvin-Benson cycle. Not all functional genes of the TCA cycle are present; there are none for the conversion of 2-oxoglutarate to succinyl-CoA. As an alternative to the TCA cycle, *Nostoc* sp. strain MS1 possesses genes encoding 2-oxoglutarate decarboxylase (OGDC) and succinic semialdehyde dehydrogenase (SSADH), which convert 2-oxoglutarate to succinic acid via succinic semialdehyde. This strain also harbors a gene that converts malate to pyruvate and produces NADPH.

**Figure 3:**
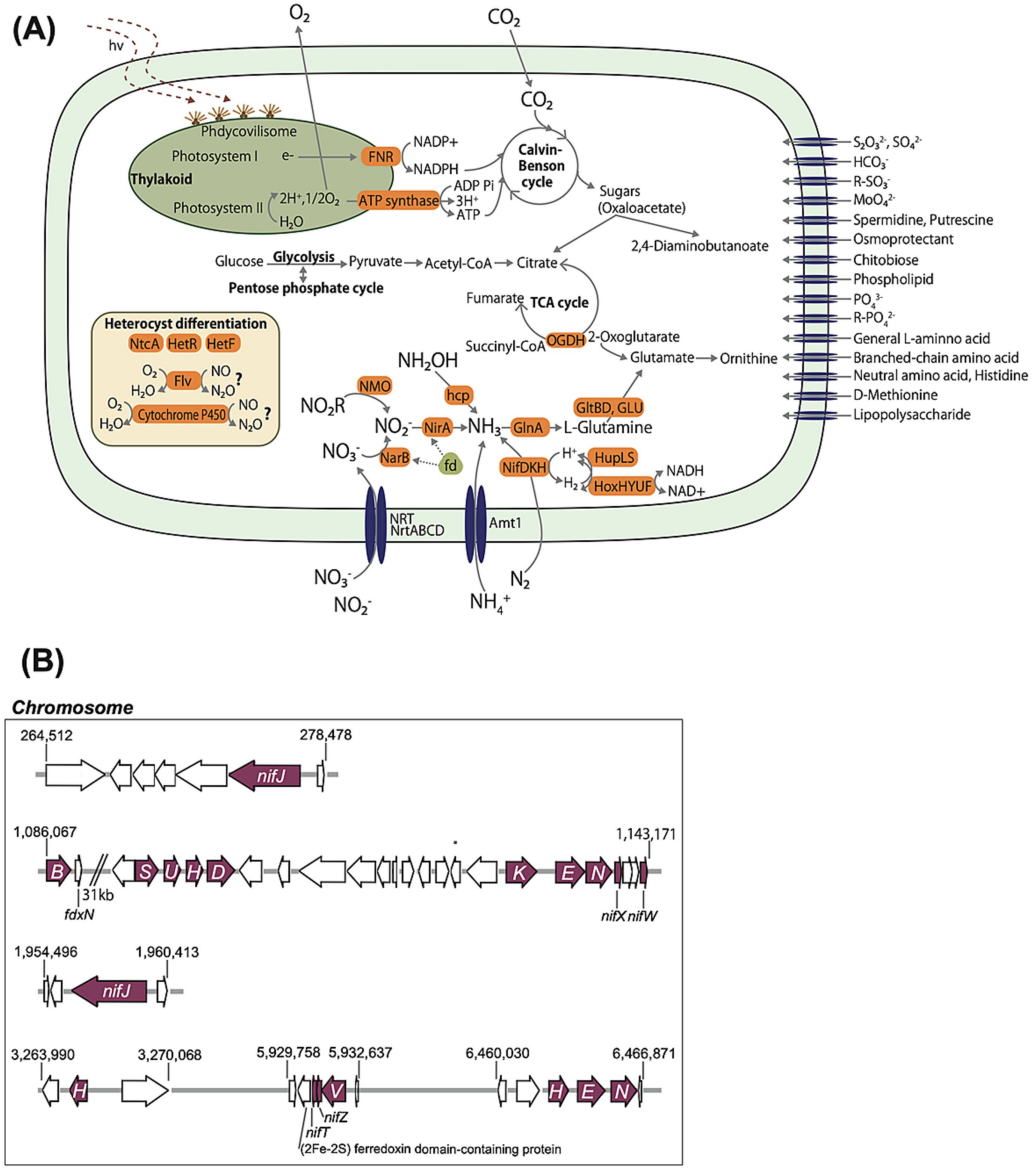
**(A)** Cell illustration highlighting metabolic features constructed from the annotation of the *Nostoc* sp. strain MS1 genome. The acronyms shaded in orange indicate enzymes. **(B)** Key nitrogenase gene loci in the genome. Amt1, ammonia transporter 1; Flv, photosynthetic flavodiiron; FNR, ferredoxin-NADP^+^ reductase; GlnA, glutamine synthetase; GltBD, glutamate synthase; GLU, glutamate synthase; hcp, hydroxylamine reductase; HetF, heterocyst differentiation protein; HetR, heterocyst differentiation control protein; HoxHYUF, cytoplasmic heteromultimeric reversible enzyme; HupLS, uptake hydrogenase; MD, NADP^+^-dependent malate dehydrogenase; NarB, nitrate reductase; NifDKH, nitrogenase iron protein; NirA, ferredoxin nitrite reductase; NMO, nitronate monooxygenase; NtcA, global nitrogen regulator; OGDC, 2-oxoglutarate decarboxylase; SSADH, succinic semialdehyde dehydrogenase.

The genome harbors *narB*, *nirA*, and *nifDKH* genes for nitrogen cycling, which encode proteins for assimilatory nitrate reduction from NO_3_^−^ to NO_2_^−^, from NO_2_^−^ to NH_3_, and for nitrogen fixation from N_2_ to NH_3_, respectively (Figure 3). The *nif* gene cluster is arranged into one operon (Figure 3B). Additionally, the strain possesses ammonium (*amt*) and nitrate/nitrite transporter genes (*nrt*). On the contrary, no homologs to the nitrogenase gene encoding vanadium (V)-nitrogenase (vnf), dissimilatory nitrite reductase gene by periplasmic cytochrome *c* (*nirAH*), and denitrifying genes, including N_2_O reduction (*nosZ*), were found in the genome. A regular gene set for the glutamine synthetase-glutamate synthase pathway is present. Based on the KEGG database, the gene groups relevant to nitrogen conversion are identical to those of other canonical *Nostoc* spp. (Supplementary Figure S3).

*Nostoc* sp. strain MS1 possesses *hupL*, *hupS*, and *hoxHYUF* (Figure 3A), encoding hydrogenases capable of coupling with O_2_ produced from photosynthesis. The genome also suggests the presence of the genes *ntcA*, *hetR*, and *hetF* for heterocyst differentiation, in which non-dividing cells perform nitrogen fixation in the absence of nitrogen under aerobic conditions. The strain MS1 harbors the genes *flv1A*, *flv1B*, *flv3A*, and *flv3B*, which encode the heterocyst-localized flavodiiron proteins that participate in the reduction of O_2_ (O_2_ → H_2_O) and nitric oxide (NO → N_2_O) (Ermakova et al., 2013). These gene sequences are more than 90% similar to those of *Anabaena* sp. PCC7120 (Ermakova et al., 2013). Strain MS1 harbors a cytochrome P450 (CYP55)-like protein that facilitates nitric oxide reduction to N_2_O, with approximately 30% similarity to that of the green microalga *Chlamydomonas reinhardtii* (Burlacot et al., 2020). The use of Pathogen Finder (ver.1.1) (Cosentino et al., 2013) to detect pathogenicity confirmed the absence of genes responsible for the production of microcystin and other toxins.

### The capability of N_2_O uptake measured by ^15^N-labeled N_2_O

**Figure 4:**
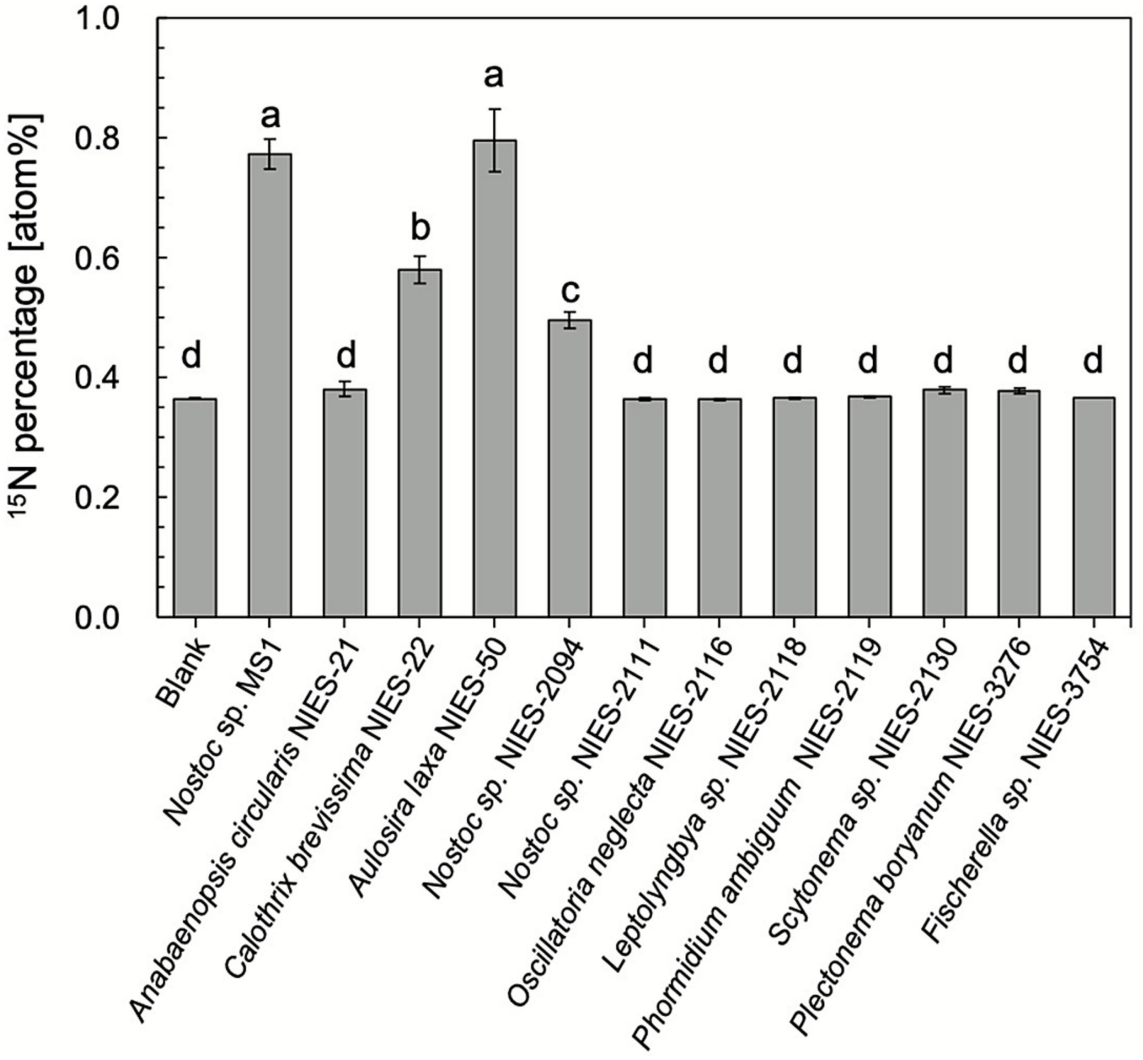
Percentage of ^15^N-labeled N_2_O taken up by cyanobacteria after 12 days of incubation. The atmospheric ^15^N percentage was approximately 0.366%. The error bars represent standard deviations (*n* = 3), and significant differences are shown with letters.

**Figure 5:**
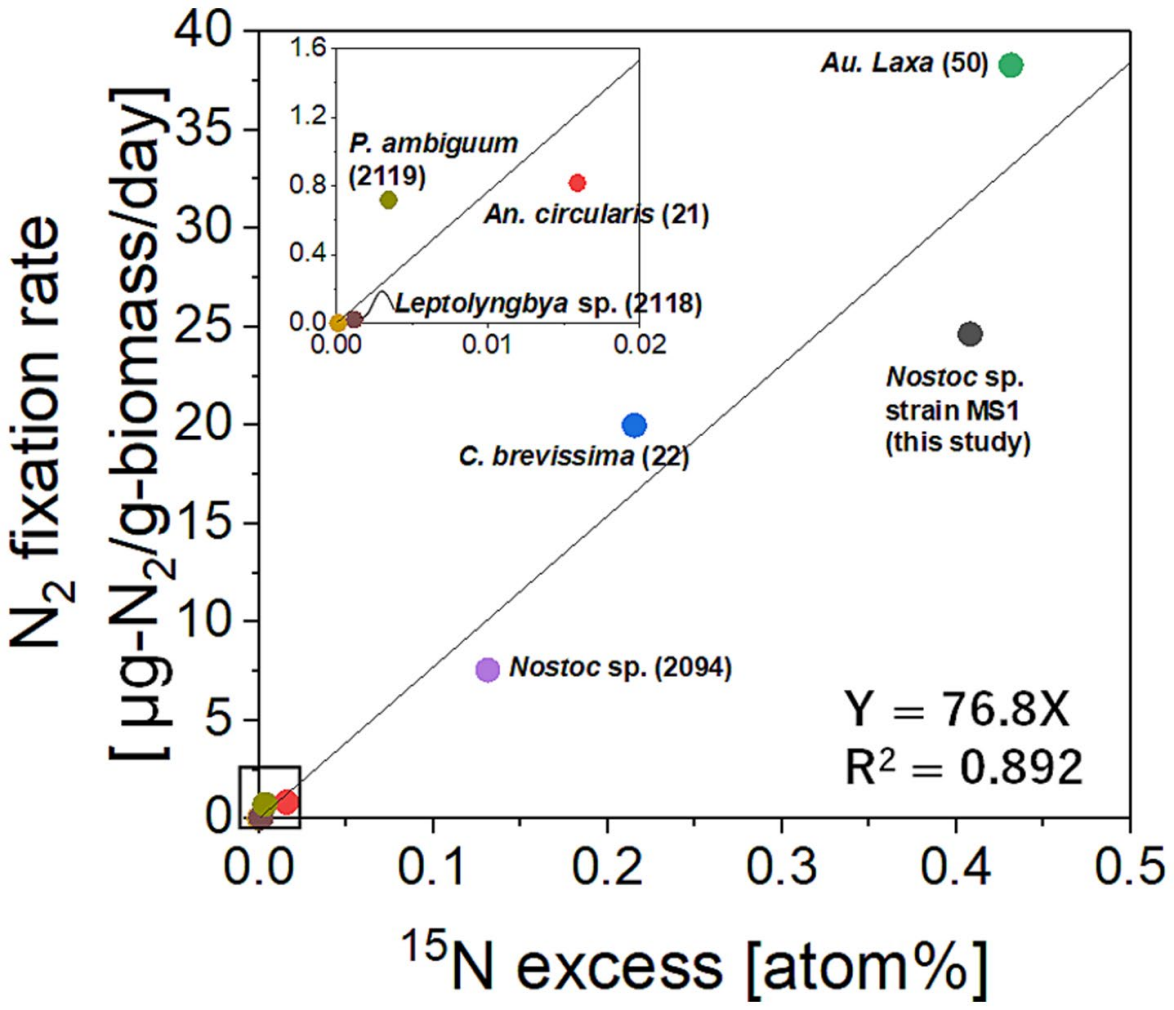
Correlation of the amount of ^15^N in cyanobacterial cells with N_2_ fixation rate by nitrogenase. The numbers in parentheses denote the NIES No. of each strain.

N_2_O fixation potentials of *Nostoc* sp. strain MS1 and other freshwater cyanobacteria (Table 1) were evaluated by supplying ^15^N-labeled N_2_O [^46^N_2_O (^15^N^15^N^16^O)]. The ^15^N percentages and weights in cyanobacterial cells after 12 days are summarized in Figure 4. *Nostoc* sp. strain MS1, *Aulosira laxa*, *Calothrix brevissima,* and *Nostoc* sp. (NIES-2094) had ^15^N uptake comparable to that originally from ^15^N-labeled N_2_O into their cells, with the final levels considerably higher than the natural abundance level. In particular, the ^15^N percentages of *Nostoc* sp. strain MS1 and *Aulosira laxa* were higher than those of the others. In contrast, the remaining tested cyanobacteria did not exhibit ^15^N uptake under batch conditions, suggesting differentiation even within the strain level. The increase in ^15^N percentage by N_2_O uptake is correlated with the nitrogen-fixing rate attained by an acetylene reduction assay (Figure 5). Although an *in vitro* study using the purified nitrogenase was not performed, the positive correlation between nitrogen uptake rate and ^15^N incorporation provides qualitative evidence for the implication of nitrogenase.

### Effect of O_2_ on N_2_O fixation by *Nostoc* sp. strain MS1

**Figure 6:**
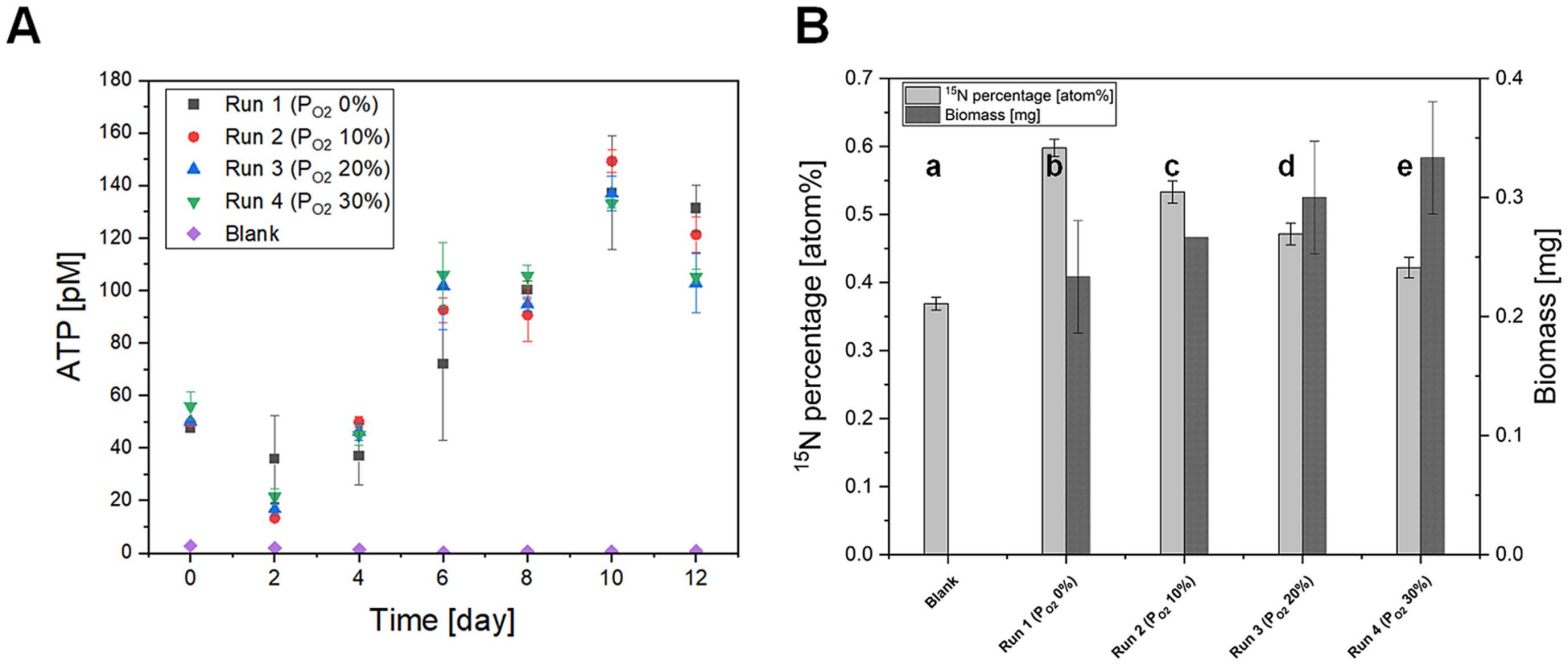
Effect of oxygen on **(A)** net ATP production and **(B)** ^15^N fixation by *Nostoc* sp. strain MS1 at the end of the experiment (12 days). The blank run contained no cyanobacterial cells. The error bars represent standard deviations (*n* = 3). Each lowercase letter denotes a statistical difference.

The effect of O_2_ on the percentage of ^15^N, derived from ^15^N-labeled N_2_O, in *Nostoc* sp. strain MS1 was investigated with four runs at different O_2_ partial pressures (P_O2_) of 0% (Run 1), 5% (Run 2), 20% (Run 3), and 30% (Run 4) (Figure 6). ATP concentration increased with no statistically significant changes between different P_O2_ in the vials (Runs 1 to 4; Figure 6A). It should be noted that the ATP concentration of Run 1–4 on day 0 and day 12 may have been influenced by carryover from the inoculum and by changes in the growth phase, respectively. Regardless of the applied P_O2_, the ^15^N percentages at the end of the experiment (12 days) exceeded the natural ^15^N abundance (Figure 6B). This result indicated that *Nostoc* sp. strain MS1 incorporated N_2_O even in the presence of O_2_, a distinct behavior from dissimilatory nitrogen reduction, which is strongly regulated by O_2_ (Klawonn et al., 2015). The ^46^N_2_O percentages in the cells decreased with increasing P_O2_, suggesting that O_2_ inhibited N_2_O uptake. The increase in O_2_ partial pressure enhanced biomass production (Figure 6B), indicating that O_2_ respiration became energetically more favorable, thereby decreasing cellular dependence on N_2_O uptake.

### Competitive effect of nitrogen on N_2_O uptake by *Nostoc* sp. strain MS1

**Figure 7:**
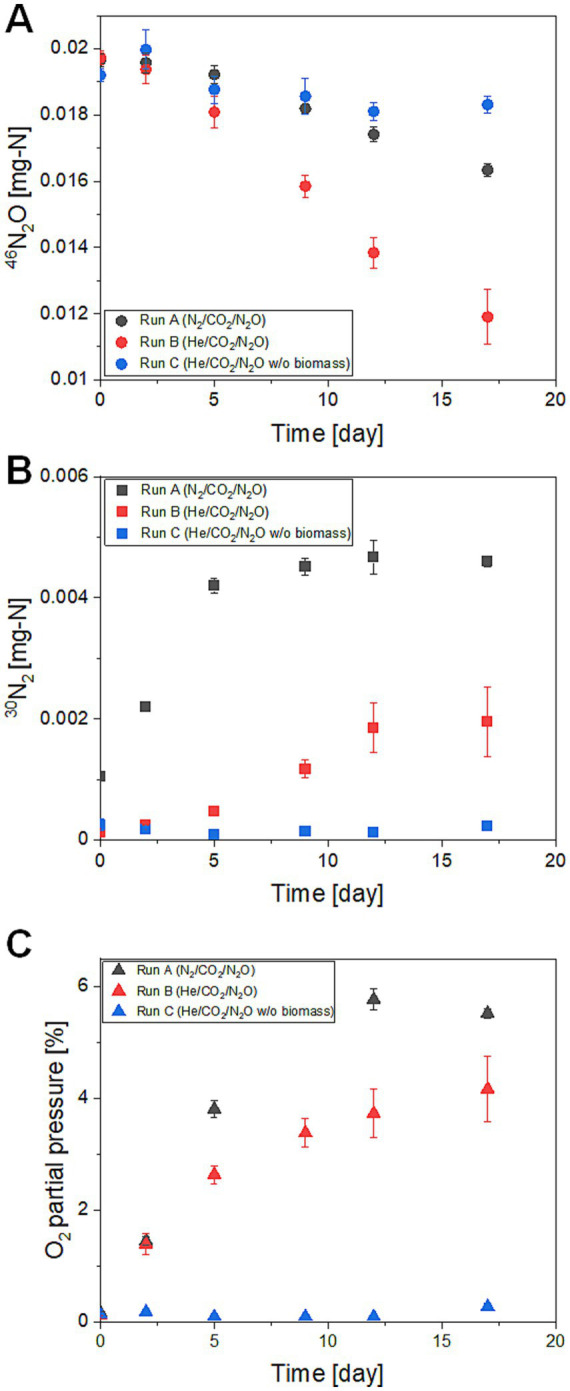
Effects of the headspace gas compositions on **(A)** ^46^N_2_O conversion, **(B)** ^30^N_2_ emitted to the headspace, and **(C)** the increase in O_2_ partial pressure in the headspace.

The N_2_O uptake performance of *Nostoc* sp. strain MS1 in the presence (Run A) or absence (Run B) of nitrogen was investigated. The incubation with N_2_ as the main headspace gas (Run A) resulted in green biomass, whereas the incubation with He and N_2_O headspace (Run B) resulted in yellow-green biomass (Supplementary Figure S4). The effect of headspace gas composition on ^46^N_2_O conversion, ^30^N_2_ emission, and O_2_ partial pressure is shown in Figure 7. The presence of N_2_ and N_2_O (Run A) retarded ^46^N_2_O consumption by 60% by day 17, compared with the consumption in the absence of N_2_ (Run B) (Figure 7A). The linear approximation of the ^46^N_2_O decreasing trends indicated that the ^46^N_2_O consumption rate in Run A was slowed by approximately 62% [4.87 × 10^1^ μg-N/g-cell/day (Run A) vs. 1.27 × 10^2^ μg-N/g-cell/day (Run B)], indicating competition for reducing equivalents between N_2_O and N_2_ assimilation. As shown in Figure 7B, Runs A and B (in the presence and absence of N_2_) emitted ^30^N_2_ gas, whereas Run C (the absence of *Nostoc* sp. strain MS1) did not; this trend followed the P_O2_ increase (Figure 7C). The results indicate that, interestingly, *Nostoc* sp. strain MS1 converted N_2_O into N_2_, followed by nitrogen fixation. The analysis of ^15^N contents in *Nostoc* sp. strain MS1 revealed that biomass in Run B had a remarkably increased ^15^N atom% (Supplementary Figure S5A). The ^15^N content in Run A slightly increased and was statistically different from the ^15^N natural abundance (0.366%). The amount of fixed nitrogen was the highest in Run A, 1.7 times higher than that in Run B (Supplementary Figure S5B).

**Figure 8:**
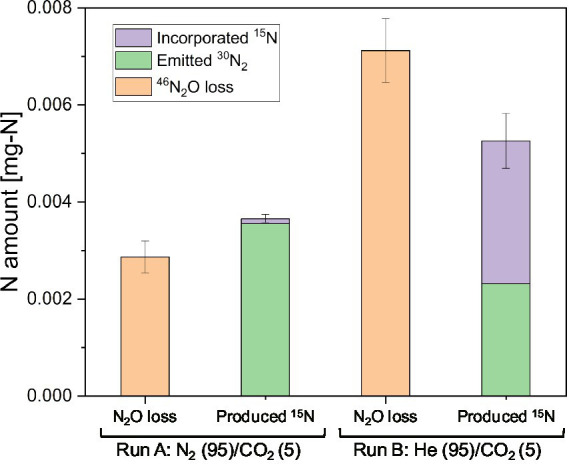
Fate of ^15^N_2_O added to the runs with a headspace of He/CO_2_ or N_2_/CO_2_. N_2_O loss was estimated by the decrease in headspace ^46^N_2_O concentration. The error bars denote standard deviations (*n* = 3).

The amount of N_2_O and the nitrogen composition in biomass from Runs A and B are summarized in Figure 8. The sum of the produced and fixed ^30^N_2_ accounted for 74% of the consumed N_2_O in Run B [He(95%)/CO_2_(5%)]. In contrast, the produced and fixed ^30^N_2_ accounted for 127% of the consumed N_2_O in Run A [N_2_(95%)/CO_2_(5%)]. Approximately half of the N_2_O was fixed by *Nostoc* sp. strain MS1 in Run B, whereas Run A emitted mostly ^30^N_2_, derived from ^46^N_2_O, to the headspace.

## Discussion

### Primary achievements and implications

Many freshwater cyanobacteria possess nitrogenase and biocatalytically convert N_2_ gas to ammonia. However, the N_2_O conversion by cyanobacteria in freshwater environments has received less attention. Whereas an *in vitro* study using purified nitrogenase demonstrated the potential involvement of freshwater bacteria in N_2_O conversion (Jensen and Burris, 1986), a ^15^N tracer study using biomass collected from freshwater ponds showed ^15^N fixation (Si et al., 2023). Yet, the pure culture-based physiology of freshwater cyanobacteria was poorly characterized. We herewith demonstrate that the freshwater cyanobacterium *Nostoc* sp. strain MS1, isolated from a nitrogen-contaminated river, anaerobically fixed the highly potent greenhouse gas N_2_O via N_2_ in the presence of excess N_2_O. The use of ^15^N tracers demonstrated that some freshwater cyanobacteria can anaerobically take up N_2_O, and likely contribute to an N_2_O sink. The riverine N_2_O emissions were estimated at 0.29 Tg-N/year in 2016, mainly from denitrification in river systems and nitrification in estuaries and reservoirs (Maavara et al., 2019). Some N_2_O produced via nitrification and denitrification may be consumed by freshwater bacteria, which have been identified as potential N_2_O sinks, e.g., the genera *Fischerella*, *Pegethrix*, and *Methylomonas* (Si et al., 2023). Our study adds a new finding: some members of the genera *Nostoc* and the proximal *Anabena* in rivers and estuaries mediate N_2_O assimilation.

This study demonstrated that *Nostoc* sp. strain MS1 could assimilate N_2_O even in the presence of O_2_, although its uptake efficiency was notably reduced. This work paves the way for a better understanding of N_2_O-related metabolism in ecosystems and for mitigating N_2_O emissions from freshwater and terrestrial environments by using freshwater cyanobacteria.

### Use of cell sorting to isolate a cyanobacterium

*Nostoc* sp. have been isolated mainly from cyanobacterial crusts in soil (Katoh et al., 2012; Hirose et al., 2016). Isolation from freshwater environments has been challenging, and our study demonstrated an effective combination of biomass enrichment and cell sorting by targeting autofluorescence and beadlike filamentous morphology. Detecting cells with high autofluorescence from chlorophyll enables sorting cyanobacteria using a fluorescence-activated cell sorter system, as previously reported (Azevedo et al., 2011; Zhou et al., 2018). A cell sorter also provides the advantage of separating bacterial cells based on the intensity of forward- and side-scattered lights, reflecting the characteristics of their cell size and intracellular complexity structures. Single-species microcolonies formed by nitrifying bacteria have been successfully separated using a cell sorter, resulting in pure cultures (Ushiki et al., 2013; Fujitani et al., 2014; Fujitani et al., 2015). An analogous strategy was successfully used to isolate *Nostoc* sp. strain MS1 based on its chain of moniliform filaments (Supplementary Figure S2) and chlorophyll autofluorescence. The use of cell sorting for freshwater cyanobacteria paves the way for acquiring more diversified cyanobacteria with unique functions, followed by a better understanding of their physiologies.

### Phylogeny and metabolic potential of *Nostoc* sp. strain MS1

Taxonomic analysis based on the 16S rRNA gene revealed that the isolate is taxonomically assigned to the genus *Nostoc*, with the highest proximity to *Nostoc* sp. CAVN2 and *Nostoc* sp. NIES-2111 in the Nostocaceae family (identity >99%) (Figure 2). *Nostoc* spp. have been previously isolated from terrestrial environments (Katoh et al., 2012; Hirose et al., 2016). Genome analysis revealed the absence of an N_2_O reductase homolog, corroborated by endpoint PCR using the primers for clade I (Henry et al., 2006) and clade II (Jones et al., 2013) *nosZ* genes. Our follow-up *in silico* investigation did not confirm the possession of the clade III *nosZ* gene (He et al., 2025). Therefore, N_2_O uptake was likely mediated, not by dissimilatory N_2_O reduction, but by assimilatory N_2_O uptake associated with nitrogenase activity. The genome also revealed that *Nostoc* sp. strain MS1 harbors MoFe-dependent nitrogenase (*nifDKH*) but not the vanadium-dependent nitrogenase (*vnf*). The absence of *vnf* in *Nostoc* sp. strain MS1 makes this strain different from other *Nostoc* spp. (Nelson et al., 2019) and *Anabaena* sp. strain ATCC29413 (Böhme, 1998), which is phylogenetically close to the genus *Nostoc*. Strains that harbor *narB* and *nirA* genes (Figure 3) may obtain ammonia via assimilatory nitrate reduction. The incomplete gene set for the TCA cycle in *Nostoc* sp. strain MS1 is also observed in other *Nostoc* spp. (Zhang and Bryant, 2011). The genome of *Nostoc* sp. strain MS1 reveals a truncated TCA shunt by OGDC and SSADH, which is fully present in most cyanobacterial genomes (Zhang and Bryant, 2011). Instead, the gene encoding NADP^+^-dependent malate dehydrogenase is present in strain MS1 (Driscoll and Finan, 1997), providing pyruvate from the incomplete TCA cycle. The holistic view of the central metabolic pathway supports *Nostoc* sp. strain MS1 as a cyanobacterium grown in freshwater environments.

Heterocyst generation potentially prevents O_2_ exposure from decreasing nitrogenase activity. Heterocysts deposit glycolipid and polysaccharide layers onto the cell wall, limiting O_2_ entry from the cell exterior. The presence of *hetR* and *ntcA* genes could regulate heterocyst development and sense nitrogen limitation (Herrero and Flores, 2019). The absence of the known denitrifying genes, *nir* (NO_2_^−^ → NO), *nor* (NO → N_2_O), and *nos* (N_2_O → N_2_), suggests that strain MS1 is not an N_2_O source via denitrification but is instead an N_2_O sink.

Nevertheless, the possibility of *Nostoc* sp. strain MS1 as an N_2_O source remains. The strain harbors *flv* and cytochrome P450 (CYP55)-like protein (Figure 3), and therefore it could reduce nitric oxide to N_2_O, which, contradictorily, suggests that strain MS1 is an N_2_O source. Flv3B, localized in the heterocyst of *Anabaena* sp., reduces molecular oxygen to water, preventing the nitrogenase in the heterocyst from oxidative damage (Ermakova et al., 2013). Given the high similarities of *flv1B* and *flv3B* gene sequences in *Nostoc* sp. strain MS1 to *Anabaena* sp. (identity >90%), strain MS1 could reduce NO to N_2_O in the light. In contrast, CYP55, found in a green alga *C. reinhardtii*, was found to reduce nitric oxide to N_2_O in the dark (Burlacot et al., 2020). Despite the low gene sequence similarity of CYP55 between *Nostoc* sp. strain MS1 and *C. reinhardtii* (<30%), strain MS1 has the whole sequence for a CYP55-like protein. We acknowledge that this study only used N_2_O as a representative of nitrogen oxides; therefore, further investigation is required to confirm the function and activity of the CYP55-like protein in *Nostoc* sp. strain MS1.

### *Nostoc* sp. as a N_2_O-fixing cyanobacterium

This study hypothesized that freshwater environments harbor cyanobacteria capable of taking up N_2_O as a nitrogen source. The use of ^15^N-labeled N_2_O (^46^N_2_O) supported our hypothesis; *Nostoc* sp. strain MS1 assimilated N_2_O, as did some other cyanobacteria affiliated with the genera *Nostoc*, *Aulosira*, and *Calothrix* (Figure 4). Moreover, this study is the first to demonstrate the positive correlation between N_2_O uptake and nitrogenase activity in cyanobacteria (Figure 5), implicating nitrogenase in N_2_O fixation. Reportedly, nitrogenase actively participates in N_2_O conversion *in vitro* (Repaske and Wilson, 1952; Rivera-Ortiz and Burris, 1975; Jensen and Burris, 1986), but no report has provided *in vivo* evidence for N_2_O fixation mediated by nitrogenase in cyanobacteria. Although marine cyanobacterial strains *Trichodesmium* and *Crocosphaera* fix N_2_O under laboratory conditions (Farias et al., 2013), no confirmation of nitrogenase activity at the isolate level is provided.

Our ^15^N tracer study demonstrated that the isolated freshwater cyanobacterium *Nostoc* sp. strain MS1 directly uses N_2_O as a nitrogen source when N_2_ is absent. In addition to N_2_O uptake, more diversified freshwater cyanobacteria are likely able to fix N_2_O, as shown in Figure 4. The data collectively and strongly indicate that some cyanobacterial species consume N_2_O in freshwater environments. The potential reason for the difference with or without N_2_O fixation function remains unclear and warrants further investigation. Other suitable environmental conditions for N_2_O fixation by freshwater cyanobacteria should be thoroughly investigated.

### O_2_ inhibition of N_2_O fixation by *Nostoc* sp. strain MS1

Dissimilatory N_2_O reduction mediated by Nos functions is an N_2_O sink. The downside of Nos as an N_2_O sink is its inactivation by O_2_, which reduces effectiveness in decreasing N_2_O emissions and preventing global warming (Pomowski et al., 2011). Alternative N_2_O consumption pathways that tolerate O_2_ exposure are required. The O_2_ resistance of nitrogenase-mediated N_2_O fixation is crucial to consider when attempting to reduce N_2_O emissions under aerobic and hypoxic conditions. Although N_2_O assimilation by *Nostoc* sp. strain MS1 was substantially decreased in the presence of O_2_ (Figure 6B), the strain produced more energy with O_2_ present and instantaneously used ATP, putatively resulting in higher biomass (Figures 6A,B). This offset may provide another advantage to *Nostoc* sp. strain MS1. The inhibition of N_2_O fixation by O_2_ has been demonstrated in previous work, in which *Pseudomonas stutzeri* reduced the N_2_O fixation rate by nitrogenase at higher P_O2_ (Desloover et al., 2014). Our study demonstrated that the relative amount of N_2_O taken into the cell at 10% P_O2_ was 89% in the absence of O_2_, whereas a low O_2_ level severely affected N_2_O consumption by Nos, which was below the detection limit at the same P_O2_ (Suenaga et al., 2018). A mechanism to provide higher O_2_ resistance remains unresolved. The high tolerance of nitrogenase against O_2_ is likely because of the heterocyst present in *Nostoc* sp. strain MS1. Flavodiiron proteins (Figure 3) may also act as O_2_ scavengers (Ermakova et al., 2013). These proteins are exclusive to cyanobacteria and may provide O_2_ resistance; this will be a topic for follow-up research.

### Competitive effect of nitrogen on N_2_O fixation by *Nostoc* sp. strain MS1

It was previously unknown whether *Nostoc* sp. strain MS1 directly assimilates N_2_O into cells without producing N_2_. Our ^15^N tracer experiment revealed that *Nostoc* sp. strain MS1 converted N_2_O into N_2_, and subsequently assimilated nitrogen (Figure 8). A mechanism for N_2_O uptake by nitrogenase is under debate. N_2_O is directly fixed by *Azotobacter vinelandii* (Yamazaki et al., 1987) but is initially converted into N_2_, followed by uptake in *P. stutzeri* (Desloover et al., 2014). The ^15^N experiments in this study support the latter scenario.

Our research also indicates that the presence of N_2_ severely slows N_2_O uptake into *Nostoc* sp. strain MS1. As shown in Figure 7, N_2_ in the headspace gas decreased ^46^N_2_O consumption (Figure 7A) and enhanced ^30^N_2_ emission (Figure 7B). The decreased N_2_O consumption is likely due to the competition between N_2_O and N_2_ for nitrogenase (Rivera-Ortiz and Burris, 1975; He et al., 2025). An *in vitro* nitrogenase study of *Klebsiella pneumoniae* demonstrated that N_2_O competes with N_2_ (Jensen and Burris, 1986). Our study reveals, for the first time, that a freshwater cyanobacterium competes for N_2_O and N_2_. The competitive inhibition of N_2_O on nitrogenase was corroborated by the enhanced ^15^N_2_O uptake without nitrogen (Run B in Figure 7A). ^30^N_2_ emissions suggests unbalance rates of N_2_O conversion into N_2_ and N_2_ fixation (Figure 8). We acknowledge that the fate of the missing 26% of nitrogen remains unclear. This missing fraction may be due to inaccurate NH_4_^+^ measurements and should be thoroughly investigated.

Table 2 summarizes the N_2_O and N_2_ fixation rates of *Nostoc* sp. strain MS1, along with those of cyanobacterial strains from freshwater environments reported in previous studies. The heterotrophic N_2_O reduction rates of denitrifying bacteria harboring clade I or clade II type *nosZ* were also listed. For the strain MS1, this study reports an N_2_ fixation rate of 2.46 × 10^4^ μg-N/g-dry biomass/day, as measured by an acetylene reduction assay (Figure 5), and N_2_O fixation rates of 4.87 × 10^1^ μg-N/g-dry biomass/day (under N_2_-based condition) and 1.27 × 10^2^ μg-N/g-dry biomass/day (under He-based condition), as determined by a ^15^N tracer method (Figure 7A). The N_2_ fixation rate was the same order of magnitude as that of *Anabaena cylindrica* and *Nostoc* sp. (Hardy et al., 1973) and higher than that of environmental samples (Kubota et al., 2023; Finke and Seeley, 1978). Meanwhile, the N_2_O fixation rates measured in the presence of N_2_ and He with 100 ppm of N_2_O were 0.20 and 0.52% of the N_2_ fixation rate, respectively. The difference in fixation rate between N_2_ (acetylene) and N_2_O may have been influenced by the applied concentrations, which can be explained by the half-saturated constants for N_2_ and acetylene in nitrogenase (*K*_m_N2_ = 0.04 atm, *K*_s_acetylene_ = 0.005 atm, Supplementary Figure S6) (Hardy et al., 1973). Although this study did not resolve the concentration dependence of N_2_O fixation rates, the observed trend suggests that the N_2_ fixation potential cannot be directly extrapolated to N_2_O, given the much lower N_2_O concentrations typically found in natural environments. The heterotrophic N_2_O reduction rate is 3 and 5–6 orders of magnitude higher than the N_2_ and N_2_O fixation, respectively, indicating that N_2_O fixation is likely detectable only in oligotrophic environments where N_2_O-fixing microorganisms are abundant.

**Table 2:** Comparison of N_2_ and N_2_O fixation and heterotrophic N_2_O reduction rates.

Biomass	Rate (μg-N/g-biomass/day)	Comments	Reference
*Nostoc* sp. strain MS1	2.46 × 10^4^	Acetylene reduction rate was converted^a^	This study
*Nostoc* sp. strain MS1	4.87 × 10^1^	N_2_O consumption rate in a N_2_-based condition (100 ppm-^15^N_2_O)	This study
*Nostoc* sp. strain MS1	1.27 × 10^2^	N_2_O consumption rate in a He-based condition (100 ppm-^15^N_2_O)	This study
*Anabaena cylindrica*	1.34 × 10^4^	Acetylene reduction rate was converted^a^	Hardy et al. (1973) and Stewart et al. (1968)
*Nostoc* sp.	2.69 × 10^3^–2.42 × 10^4^	Acetylene reduction rate was converted^a^	Hardy et al. (1973), Stewart et al. (1968), and Stewart et al. (1967)
Freshwater ponds biomass	0.161	N_2_ fixation rate	Si et al. (2023)
Freshwater ponds biomass	0.0742	N_2_O fixation rate	Si et al. (2023)
*Cyanobacteria living on moss* (*Hylocomium splendens*)	6.34 ± 6.72	Converted acetylene reduction rate based on g-dry moss^a^	Kubota et al. (2023)
*Cyanobacteria living on moss* (*Pleurozium schreberi*)	2.52 ± 3.64	Converted acetylene reduction rate based on g-dry moss^a^	Kubota et al. (2023)
*Freshwater pond biomass*	2.52 × 10^1^–1.01 × 10^2^	Acetylene reduction rate was converted^a^	Finke and Seeley (1978)
*Pseudomonas stutzeri* strain DCP-1	1.68 × 10^8^	Heterotrophic N_2_O reduction rate (clade I *nosZ*)	Yoon et al. (2016)
*Azospira* sp. strain I13	4.29 × 10^7^	Heterotrophic N_2_O reduction rate (clade II *nosZ*)^b^	Suenaga et al. (2019)

a The molar conversion ratio of acetylene reduction with nitrogen fixation was applied C_2_H_2_:N_2_ = 3:1.

b The rate was converted using the 0.28 pg dry-weight per bacterial cell (Madigan and Martinko, 2006).

Together, we isolated a freshwater cyanobacterium, *Nostoc* sp. strain MS1, by enrichment and cell sorting. This cyanobacterium assimilates the greenhouse gas N_2_O via N_2_. This discovery of N_2_O fixation by a unique freshwater cyanobacterium illuminates an additional N_2_O consumption pathway that may contribute to an N_2_O sink in freshwater environments. The genome analysis of *Nostoc* sp. strain MS1 reveals metabolic potentials for nitrogen fixation, nitrogen conversion, and an O_2_-scavenging role of the heterocyst. The use of ^15^N_2_O tracer and acetylene experiments indicated that *Nostoc* sp. strain MS1 and cyanobacteria affiliated with *Calothrix*, *Aulosira*, and *Nostoc* can assimilate N_2_O, potentially by nitrogenase. The ^15^N tracer study revealed N_2_O uptake via N_2_ production by strain MS1 nitrogenase, and a declining trend when O_2_ and nitrogen were present. The effects of O_2_ and nitrogen on N_2_O uptake by these N_2_O-consuming cyanobacteria and their abundances in freshwater habitats will be systematically evaluated in future experiments.

## Data Availability

The strain MS1 is publicly available. The high-quality draft genome sequence of *Nostoc* sp. strain MS1 has been deposited as six contigs in DDBJ/EMBL/GenBank under the accession numbers AP023441–AP0023446. The BioSample accession number is SAMD00242822. The read data generated by MinION and NovaSeq have been deposited in DDBJ Sequence Read Archive (DRA) under BioProject accession number PRJDB10449 and DRA accession number DRA010693.

## Footnotes

1
https://github.com/rrwick/Porechop
