# Supplementary material for: Assimilatory N2O reduction by Nostoc sp. strain MS1 isolated from a river: insights from genome and 15N tracer analysis
Source: Front Microbiol. 2026 Mar 16;17:1759539. doi: 10.3389/fmicb.2026.1759539 (PMC13033781; doi:10.3389/fmicb.2026.1759539)
Supplement: Supplementary file 1 [file Data_Sheet_1.docx]

**Supporting information**

**Assimilatory N_2_O reduction by *Nostoc* sp. strain MS1 isolated from a river: insights from genome and ^15^N tracer analysis**

Kazumi Suda^1^, Toshikazu Suenaga^2*^, Soichiro Matsuzaki^1^, Shohei Riya^1^, Kento Ishii^3^, Manami Nomachi^3^, Hirotsugu Fujitani^1, 3^, Satoshi Tsuneda^3^, Kartik Chandran^4, 5^ and Akihiko Terada^1,5,6*^

^1^ Department of Applied Physics and Chemical Engineering, Tokyo University of Agriculture and Technology, 2-24-16 Naka, Koganei, Tokyo, 184-8588, Japan

^2^ Department of Chemical Engineering, Hiroshima University, 1-4-1 Kagamiyama, Higashi-hiroshima, Hiroshima, 739-8527, Japan

^3^ Department of Life Science and Medical Bioscience, Waseda University, Tokyo, Japan

^4^ Department of Earth and Environmental Engineering, Columbia University, New York, USA

^5^ Institute of Global Innovation Research, Tokyo University of Agriculture and Technology, 3-8-1 Harumi-cho, Fuchu, Tokyo, 185-8538, Japan

^6^ Research Division of Nutrient Management, Advanced Research Center for One Welfare, Tokyo University of Agriculture and Technology, 3-8-1 Harumi-cho, Fuchu, Tokyo 185-8538, Japan

^*^ Corresponding: A. Terada ([akte@cc.tuat.ac.jp](mailto:akte@cc.tuat.ac.jp)) and T. Suenaga ([suenagat@hiroshima-u.ac.jp](mailto:suenagat@hiroshima-u.ac.jp))

The number of pages: 13

The number of figures: 6

The number of tables: 4

**Phylogenetic analysis of the isolated cyanobacteria**

PCR was used to amplify the 16S rRNA genes. The reagent consisted of 14.4 µL nucleotide-free water, 2 µL 10× ExTaq buffer, 1.6 µL dNTP mix, 0.4 µL 10 nM forward (341f) and reverse (907r) primers, 0.16 µL ExTaq polymerase (Takara Bio, Shiga, Japan), and 1 µL extracted DNA. The PCR conditions involved initial denaturation at 95 ºC for 5 min, followed by 40 cycles of denaturation at 94 ºC for 30 sec, annealing at 55 ºC for 30 sec, extension at 72 ºC for 30 sec, ending with a final extension at 72 ºC for 2 min. The PCR amplicon was ligated into a plasmid vector (pGEM-T Easy Vector, Promega, WI) and transformed into *Escherichia coli* competent cells (Competent high DH5α, Toyobo, Osaka, Japan). After incubating the *E. coli* cells, the plasmid DNA containing the inserted DNA amplicon was extracted using a MagExtractor-Plasmid kit (Toyobo, Osaka, Japan), followed by DNA sequencing of the 16S rRNA gene fragment. The sequence was aligned, and the phylogenetic tree was constructed with the Maximum Likelihood method using MEGA (ver. 12).

**Preparation of lyophilized cell samples for ^15^N**

At the end of the batch experiment, the cell suspension was filtered through a glass filter (GF/F, GE Healthcare, Kent, UK), which had been washed with distilled water and dried at 600 ºC for 30 min. The filter, containing the cell pellet, was transferred to a screw-top plastic tube and stored at −20 ºC until analysis. The glass filter was lyophilized in a dry chamber (Eyela, Tokyo, Japan), weighed, and the ^15^N ratio (^15^N Atom%) and total nitrogen were measured by an isotope ratio mass spectrometry (IR-MS, Flash Flash2000-DELTAplus Advantage conFloI System; Thermo Fischer, Waltham, MA).

**Isolation of cyanobacteria by a cell sorter**

On the basis of the forward scatter and PerCP-Cy5.5 values, representing the cell clump (or a single cell) morphology and chlorophyll fluorescence intensity, respectively, a dot plot area (P3) was identified (**Figure 1A**). The cell aggregate morphologies in the P3 area resembled those of canonical cyanobacteria assimilating nitrogen; therefore, cells in this area were transferred to a 96-well plate using a fluorescence-activated cell sorter (FACS). The subsequent aerobic incubation did not yield a pure culture of a single cyanobacterium because a mixture of 16S rRNA genes from multiple microbes was detected (data not shown). Therefore, the cell suspensions were subjected to a second round of cell sorting using FACS to further increase purity. The cell collections in the P1 area (**Figure 1B**) were sorted into 96-well plates and incubated aerobically. The application of the second round of FACS, followed by aerobic incubation, enabled the isolation of a cyanobacterium.


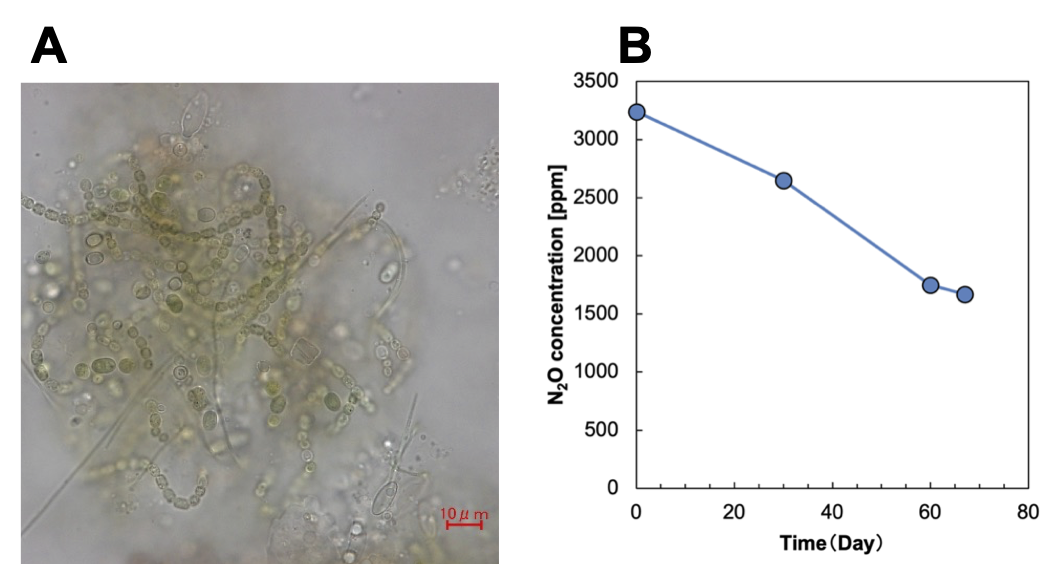


**Figure S1.** (A) The microscopic image of the enriched biomass and (B) N_2_O consumption activity during the enrichment period under the headspace condition of He (94.7%), CO_2_ (5%), and N_2_O (0.3%).


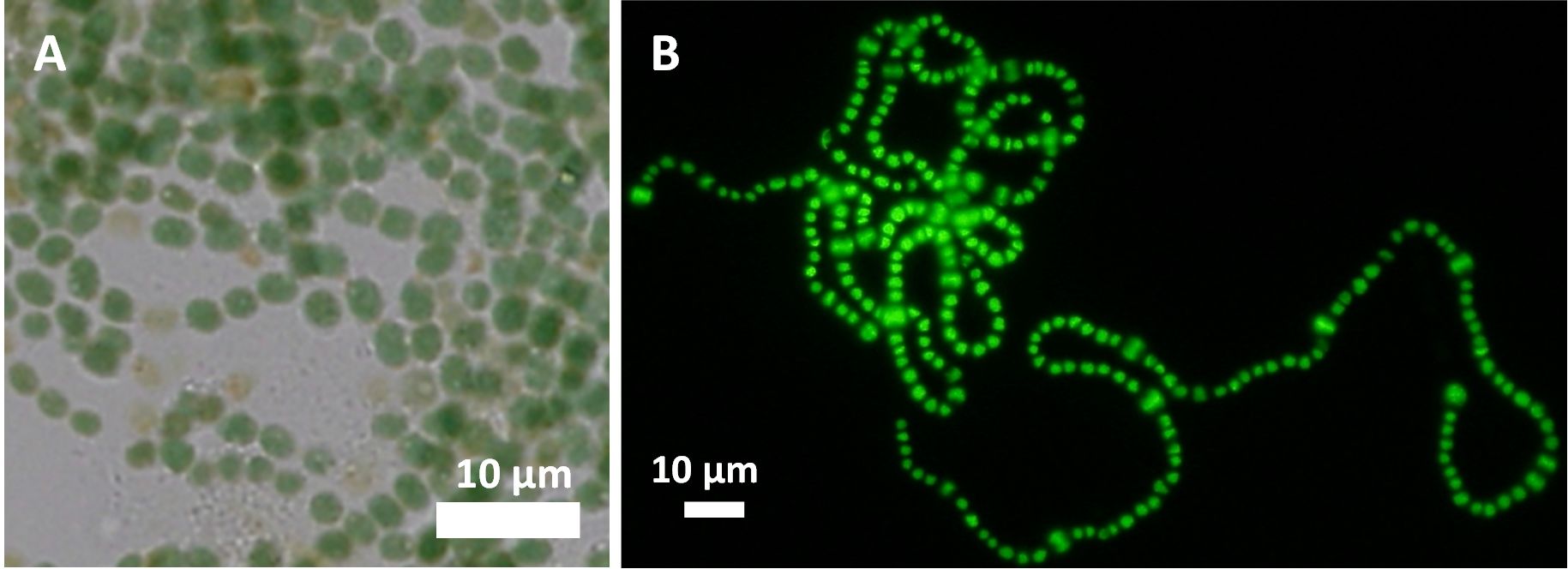


**Figure S2.** Morphological traits of the isolated cyanobacterium by (A) light microscopy and (B) epifluorescence microscopy using SYTO 9.


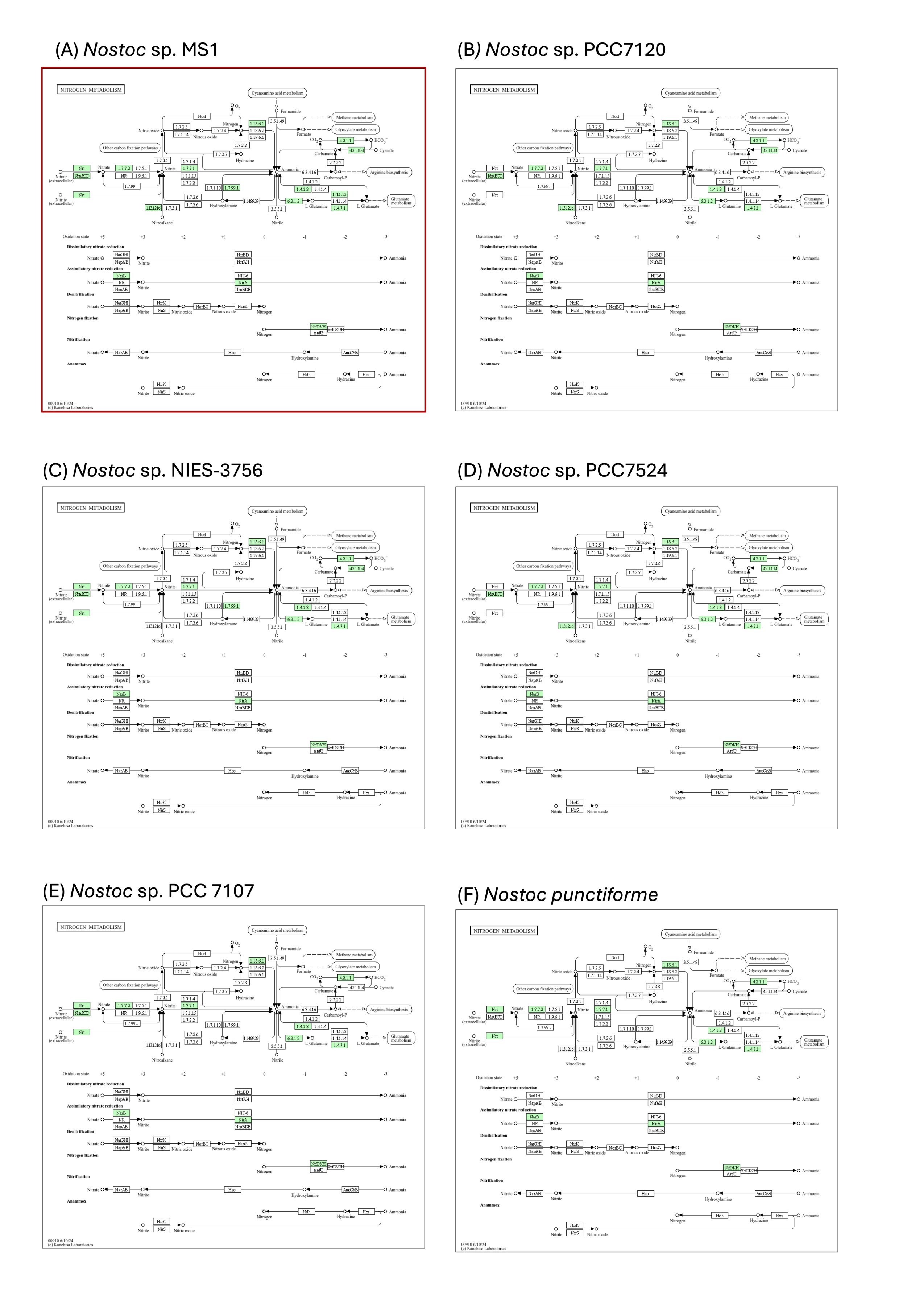


**Figure S3** A comparison of the gene groups relevant to the nitrogen metabolism of *Nostoc* sp. MS1 (A), *Nostoc* sp. PCC7120 (B), *Nostoc* sp. NIES-3756 (C), *Nostoc* sp. PCC7524 (D), *Nostoc* sp. PCC7107 (E), and *Nostoc punctiforme* (F). The reference strains have been registered in the KEGG Pathway Database (Kanehisa and Goto, 2000).

**A**

**B**

**C**

**Figure S4.** Color change in *Nostoc* sp. strain MS1 after the incubation with a headspace of mainly (A) nitrogen (Run A), (B) helium (Run B), and (C) N_2_O.

**A**

**B**

**Figure S5** (A) ^15^N atom% and (B) the amount of fixed nitrogen in *Nostoc* sp. strain MS1 biomass under each headspace gas condition. The error bars denote standard deviations (n = 3).

**
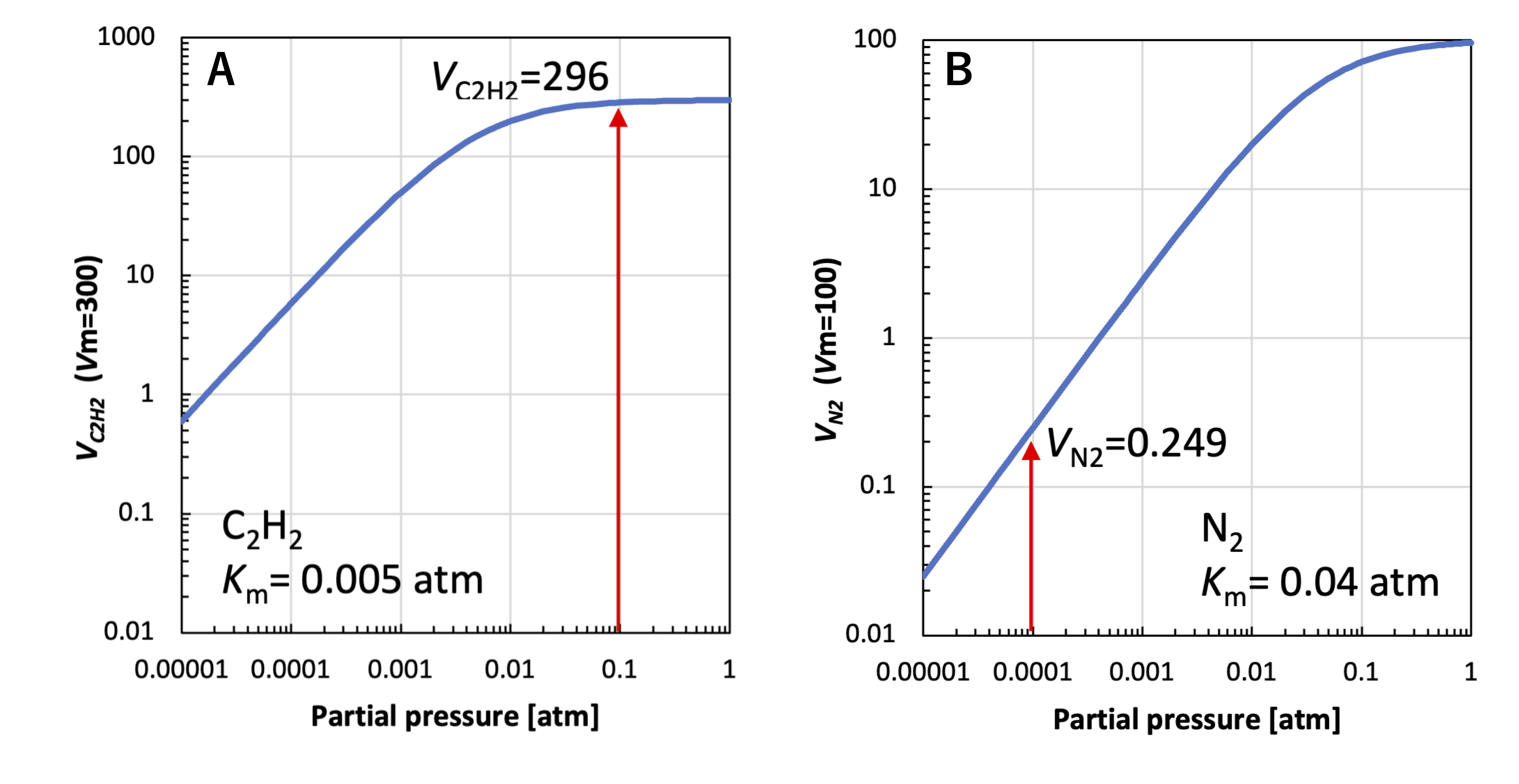
**

**Figure S6** Double-logarithmic graphs of the Michaelis-Menten equation for nitrogenase. The substrates are (A) acetylene and (B) N_2_, and *V*_m_ is set to *V*_m_C2H2_= 3×*V*_m_N2,_ which considers the molar conversion ratio of acetylene reduction with nitrogen fixation (3:1). In this study, 10% (0.1 atm) of acetylene was used for the assay and the 100 ppm (0.0001 atm) of N_2_O was applied for the ^15^N tracer method. The N_2_ fixation rate measured using the acetylene reduction assay is 96.7 (=296/3). Assuming that N_2_O follows the same manner as N_2_-nitrogenase (B), the N_2_O fixation rate would be 0.249. The differences in the N_2_ and 100 ppm-N_2_O fixation rates are 368-fold.

**Table S1** GC-FID measurement conditions for determination of ethylene concentrations

| Column temperature [ºC] | 40 |
| --- | --- |
| Injection temperature [ºC] | 180 |
| Detector temperature [ºC] | 180 |
| Carrier gas flow rate [mL/min] | 29 |
| H_2_ pressure [kPa] | 50 |
| Air pressure [kPa] | 50 |
| Column | Porapack R |

**Table S2** Composition of modified BG11_0_ medium

| Reagent | Amount |
| --- | --- |
| 1 M HEPES-NaOH | 2 mL |
| K_2_HPO_4_⋅3H_2_O | 4 mg |
| MgSO_4_⋅7H_2_O | 7.5 mg |
| CaCl_2_⋅2H_2_O | 3.6 mg |
| Citric acid | 0.6 mg |
| Ferric ammonium citrate | 0.6 mg |
| Na_2_EDTA-Mg | 0.1 mg |
| Na_2_CO_3_ | 2 mg |
| A6 | 0.1 mL |
| Vitamin B_12_ | 0.1 μg |
| Biotin | 0.1 μg |
| Thiamine HCl | 0.2 mg |
| ddH_2_O | 97.9 mL |

**Table S3** Gas chromatography-electron capture detector (GC-ECD) conditions for analysis of gaseous N_2_O concentrations

| Column temperature [ºC] | 230 |
| --- | --- |
| Injection temperature [ºC] | 80 |
| Detector temperature [ºC] | 3.2 |
| Carrier flow [kPa] | 50 |
| Make up flow [kPa] | 15 |
| Time [h] | 24 |
| Carrier gas composition | 95% Ar + 5% CH_4_ |

**Table S4** GC-mass spectrometry (GC-MS) conditions

| Injection port | Split ratio | 10 |
| --- | --- | --- |
|  | Injection volume [mL] | 200 |
|  | Temperature [ºC] | 100 |
| GC separator | Flow rate [mL/min] | 25.3 |
|  | Pressure [kPa] | 38.0 |
|  | Column flow rate [mL/min] | 2.03 |
|  | Flow velocity [cm/sec] | 56.6 |
|  | Temperature [ºC] | 50 (within 1.4 min) |
| MS detection part | Temperature at the ion source [ºC] | 200 |
|  | Temperature at the interface [ºC] | 250 |
|  | Detection voltage [kV] | 1.2 |
|  | Detection mode | SIM |

**References**

Kanehisa, M., and Goto, S. (2000). KEGG: Kyoto Encyclopedia of Genes and Genomes. Nucleic Acids Research 28, 27–30.
